# Imaging-based adipose biomarkers for predicting clinical outcomes of cancer patients treated with immune checkpoint inhibitors: a systematic review

**DOI:** 10.3389/fonc.2023.1198723

**Published:** 2023-10-17

**Authors:** Xinyu Pei, Ye Xie, Yixuan Liu, Xinyang Cai, Lexuan Hong, Xiaofeng Yang, Luyao Zhang, Manhuai Zhang, Xinyi Zheng, Kang Ning, Mengyuan Fang, Huancheng Tang

**Affiliations:** ^1^ Department of Gastroenterology, The Central Hospital of Wuhan, Tongji Medical College, Huazhong University of Science and Technology, Wuhan, China; ^2^ Zhongshan School of Medicine, Sun Yat-sen University, Guangzhou, China; ^3^ Department of Head and Neck Surgery, Sun Yat-sen University Cancer Center, Guangzhou, China; ^4^ State Key Laboratory of Oncology in Southern China, Collaborative Innovation Center for Cancer Medicine, Guangzhou, China; ^5^ Department of Ultrasound, Changsha Hospital for Maternal & Child Health Care Affiliated to Hunan Normal University, Changsha, China; ^6^ Department of Urology, Wuhan Third Hospital, Tongren Hospital of Wuhan University, Wuhan, China

**Keywords:** adipose tissue, cancer, immune checkpoint inhibitor, prognosis, radiomics

## Abstract

**Background:**

Since the application of Immune checkpoint inhibitors (ICI), the clinical outcome for metastatic cancer has been greatly improved. Nevertheless, treatment response varies in patients, making it urgent to identify patients who will receive clinical benefits after ICI therapy. Adipose body composition has proved to be associated with tumor response. In this systematic review, we aimed to summarize the current evidence on imaging adipose biomarkers that predict clinical outcomes in patients treated with ICI in various cancer types.

**Methods:**

Embase and PubMed were searched from database inception to 1st February 2023. Articles included investigated the association between imaging-based adipose biomarkers and the clinical outcomes of patients treated with ICI. The methodological quality of included studies was evaluated through Newcastle- Ottawa Quality Assessment Scale and Radiomics Quality Score tools.

**Results:**

Totally, 22 studies including 2256 patients were selected. Non-small cell lung cancer (NSCLC) had the most articles (6 studies), followed by melanoma (5 studies), renal cell carcinoma (RCC) (3 studies), urothelial carcinoma (UC) (2 studies), head and neck squamous cell carcinoma (HNSCC) (1 study), gastric cancer (1 study) and liver cancer (1 study). The remaining 3 studies investigated metastatic solid tumors including various types of cancers. Adipose biomarkers can be summarized into 5 categories, including total fat, visceral fat, subcutaneous fat, intramuscular fat and others, which exerted diverse correlations with patients’ prognosis after being treated with ICI in different cancers. Most biomarkers of body fat were positively associated with survival benefits. Nevertheless, more total fat was predictable of worse outcomes in NSCLC, while inter-muscular fat was associated with poor clinical benefits in UC.

**Conclusion:**

There is relatively well-supported evidence for imaging-based adipose biomarkers to predict the clinical outcome of ICI. In general, most of the studies show that adipose tissue is positively correlated with clinical outcomes. This review summarizes the significant biomarkers proven by researches for each cancer type. Further validation and large independent prospective cohorts are needed in the future. The protocol of this systematic review has been registered at the International Prospective Register of Systematic Reviews (http://www.crd.york.ac.uk/PROSPERO, registration no: CRD42023401986).

## 1 Introduction

Immune checkpoint inhibitors (ICI) have revolutionized the clinical survival of patients with advanced cancers. Since the introduction of ICI in melanoma in 2011, the 5-year survival rates could approach 35% to 40% in metastatic melanoma with an average life expectancy ranging from six to twelve months before (1). For non-small cell lung cancer (NSCLC), accounting for 80-90% of primary lung cancer (2), the approval of ICI in 2015 increased the 5-year survival rate from less than 10% to more than 30% (3). Currently, immunotherapy, particularly ICI, is an attractive and viable treatment option for various cancer types (4). ICI achieves clinical success by inhibiting the immune checkpoints between differentiation 8 (CD8) T lymphocytes and tumorigenic cells, with the programmed cell death protein 1 (PD-1), the programmed death-ligand 1 (PD-L1) and CTLA-4 being the most well-studied checkpoints (5). Despite its success, treatment response to ICI varies greatly among patients, with some experiencing adverse events and minimal benefits. For instance, only 4% of patients with NSCLC showing remission, defined as responding to ICI, were still alive after 4.5 years. In addition, it is universally acknowledged that immunotherapy is costly and can lead to numerous adverse events, such as colitis, diarrhea and polyarthritis (6). Therefore, identifying patients who are likely to benefit from ICI treatment is of great significance (7).

Several biomarkers have been identified to predict the response of ICI, while accurately predicting clinical outcomes remains a challenge. Biomarkers related to ICI treatment are often collected from tissue samples and include inflammatory cytokines, tumor-infiltrating lymphocytes, mutation variants, and levels of PD-L1 or CTLA-4 (8). Despite their association with a favorable response for the above biomarkers, their predictive power and feasibility are still uncertain. For example, the adoption of the percentage of tumor cells expressing PD-L1, a commonly used biomarker based on the mechanism of ICI (9), remained controversial regarding its reliability and disability of reflecting dynamic PD-L1 expression (10, 11). A large meta-analysis also revealed that PD-L1 expression status alone is insufficient for determining which patients should receive PD-1 or PD-L1 blockade therapy (12). Current predictive factors do not fully meet the needs of clinical prognosis prediction for ICI.

Body composition, measured by imaging method, was reported to be significantly associated with the clinical benefit of ICI (13). Visceral adipose tissue, subcutaneous adipose tissue, intra-muscular adipose tissue and total body fat tissue, are widely studied body composition. Adipose tissue is implicated in tumorigenesis and progression, while the “obesity paradox” suggests that obese tumor patients have better survival outcomes during treatment (14). Adipose tissue in visceral and subcutaneous have different origins, which may determine the functional heterogeneity in tumors (15). Recent studies have investigated the association between adipose composition and immunotherapy efficacy. Takenaka et al. reported lower visceral fat was significantly associated with poor disease control in head and neck squamous cell carcinoma (HNSCC) (16). Sabel et al. found higher visceral fat distance predicted poor survival of patients with melanoma (17). The results in different cancers and the distinctive biomarkers applied to even the same tissue vary. Thus, there is a need to identify specific adipose biomarkers to predict clinical outcomes in specific cancers.

In this systematic review, we aimed to summarize the ability of different imaging-based adipose biomarkers to predict the clinical outcomes after ICI treatment. The patients with any malignancy treated with ICI were the targeted population. Investigated predictors are adipose biomarkers and models deriving from imaging. Investigated clinical outcomes include therapy response, progression-free survival (PFS), overall survival (OS) and tumor remission. The quality of image mining cohort studies was assessed by Newcastle-Ottawa Quality Assessment Scale (NOS) and radiomics quality scoring (RQS) tools.

## 2 Materials and methods

### 2.1 Protocol registration

The systematic review was performed in accordance with the Preferred Reporting Items for Systematic Reviews and Meta-analyses (PRISMA) guidelines (18). The protocol of this systematic review has been registered at the International Prospective Register of Systematic Reviews (http://www.crd.york.ac.uk/PROSPERO, registration no: CRD42023401986).

### 2.2 Search strategy

The electronic databases including PubMed and Embase were searched to identify relevant studies from inception to February 2023 with the limitations of English language and human subjects using a combination of relevant free text terms and controlled vocabulary (MeSH or EMTREE terms). The details of search strategies are presented in **Supplementary Tables 1**, **2**.

### 2.3 Eligible criteria

Studies were included if they were prospective and retrospective observational studies that reported the associations between imaging-based adipose biomarkers and clinical outcomes of cancers treated with ICI. The adipose biomarkers in the observational study needed to be measured by standardized and validated software analyzing the medical images, such as CT and MRI. The cancers also needed to be identified based on the medical records. The study population was restricted to adults aged 18 years or older. Only studies with English versions were included. Exclusion criteria were as follows: (1) non-original research such as case reports, reviews, letters, comments and meta-analyses; (2) studies without comparison by imaging-based adipose biomarkers category; (3) studies with insufficient information to evaluate the effect of imaging-based adipose biomarkers on clinical outcome of cancers. (4) Studies only reported other types of immunotherapy instead of ICI therapy. Unpublished data and studies not published in peer-reviewed journals were also excluded.

### 2.4 Study selection and data extraction

After the removal of duplicates, titles and abstracts were preliminarily screened, and full-text articles of potentially relevant studies were retrieved for further assessment of eligibility by two independent reviewers (XP and YX). Any disagreements were resolved through discussion with a senior reviewer (KN). Additionally, manual reference list searches of retrieved studies were conducted to identify additional eligible articles. Data extraction was conducted according to a predefined data extraction form by two reviewers (XP and YL) independently, and the results were further verified by a senior reviewer (KN). The following information was extracted from each study: first author and publication year, study design, study population, sample size, exposure assessment and main results. Discrepancies in data extraction were discussed and resolved by consensus among the reviewers.

### 2.5 Study quality assessment

The NOS was used to assess the quality of all retrospective studies based on three domains: selection of study groups (0-4 scores), comparability of groups (0-2 scores), and ascertainment of exposure or outcomes (0-3 scores) (19). The total scores of 0-3, 4-6, and 7-9 were considered to represent low, moderate, and high quality, respectively. In addition to the risk of bias evaluation, the 16-component RQS tool was used to assess the quality of all radiomic studies (20). Two investigators (YL and YX) independently evaluated the studies, and disagreements were resolved by consensus.

## 3 Result

**Figure 1:**
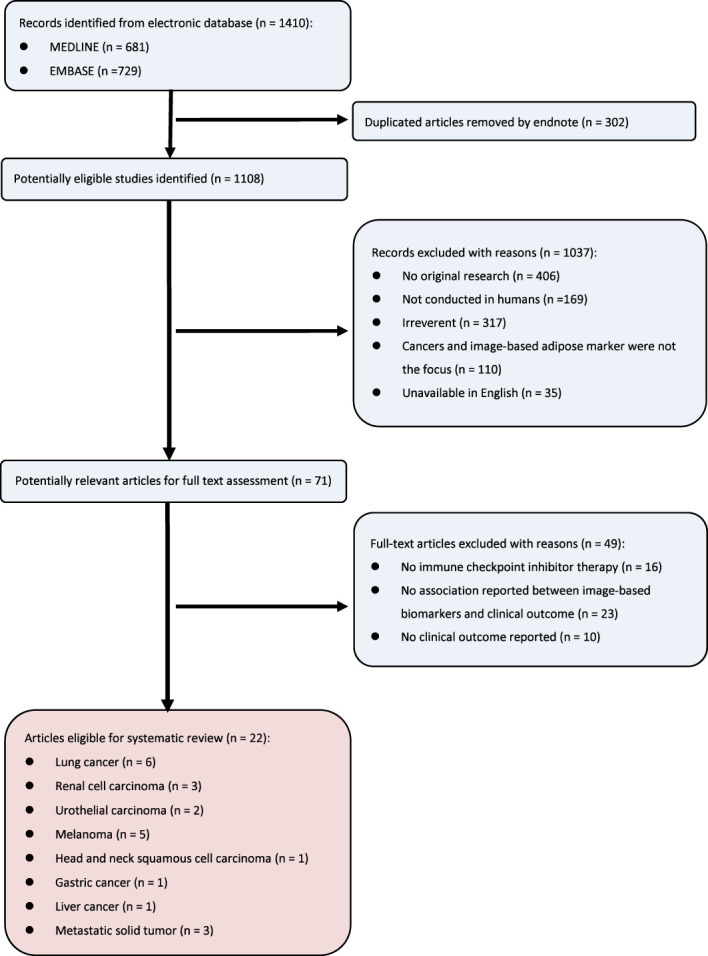
Flow diagram for study selection.

The systematic search workflow is shown in **Figure 1**. Our initial search yielded 1410 potentially relevant records from the MEDLINE and EMBASE databases. After removing 302 duplicates, 1108 articles were screened by abstract and title, and only 71 articles were retained for full-text screening. Ultimately, 22 studies were included for analysis, with 49 studies excluded (21–31).

### 3.1 General characteristics

**Table 1** displays detailed characteristics of the 22 articles included in this study. The total number of patients analyzed was 2,256, with a median sample size of 85, ranging from 18 to 287. As for the specific type of cancers, the majority of articles examined lung cancer (6 studies), followed by melanoma (5 studies), renal cell carcinoma (3 studies), urothelial carcinoma (2 studies), head and neck squamous cell carcinoma (1 study), gastric cancer (1 study) and liver cancer (1 study). The remaining 3 studies investigated metastatic solid tumors including various types of cancers. All researches were retrospective cohort studies with a median follow-up of 17.3 months. All patients received at least one ICI agent, with 12 studies using anti-PD-1/PD-L1 therapy and 4 studies using anti-CTLA4 therapy. CT was the most commonly used imaging modality, followed by PET/CT and MRI. Quality assessment using NOS and RQS screening is provided in **Supplementary Tables 3**, **4**, respectively.

**Table 1:** Characteristics of Included Studies.

Cancer(s)	Author (year)	Country	Population, total/males	Age (years)	Follow-up (months)	Method	Adipose biomarkers	Study results	ICI
Lung cancer	Popin (2019)	France	55/41	63.5	NA	PET/CT	FBM, VFM, SCFM	OS↑: FBM (Uni: HR = 0.80; p = 0.004/Multi: HR= 0.75; p = 0.006), VFM (p = 0.008), SCFM (HR = 0.75; p = 0.003)	Nivolumab (Anti PD-1)
	Minami (2020)	Japan	74/48	70	NA	L3 (CT)	IMAC, VSR, VFA	OS↑: IMAC (uni: p = 0.15/multi: HR= 0.43; p = 0.0496), others were negative; PFS: all negative	Anti PD-1/PD-L1
	Baldessari (2021)	Italy	44/26	70	8.8	Psoas muscle level (CT)	VFA, VFI, SFA, SFI, TFI, VSR, SCFM, FBM	OS: all negative	Pembrolizumab (Anti PD-1)
	Degens (2021)	Netherlands	80/46	64.9	NA	L1 (CT)	VFI, SFI	OS↓: WL characterized by decreased VFI and SFI (HR=2.39; p< 0.001);	Nivolumab (Anti PD-1)
	Nishioka (2022)	Japan	74/67	67.5	19.5	L3 (CT)	TFI	ORR ↑: decreased TFI (P = 0.005); PFS↑ : decreased TFI (uni: P = 0.037 / multi: HR = 0.34; P < 0.05);	anti-PD1/PD-L1
	Bolte (2022)	USA	92/48	64	29.6	L3 (PET/CT)	IMAC	OS negative	ICI and CTX
Renal cell carcinoma	Martini (2021)	USA	79/58	61	NA	L3 (CT)	TFI, Body composition risk score*	OS↑: TFI (p =0.001); OS↓: score (HR = 6.37; p< 0.001) PFS↑: TFI (p =0.002) PFS↓: score (HR = 4.19; p < 0.001) CB↑: TFI CB↓: score (OR= 0.23; p< 0.044);	ICI
	Aslan (2022)	Turkey	52/34	NA	11.4	L3 (CT)	SFI	OS negative	Anti PD-1
	Wang (2022)	China	251/178	55	10.1	L3 (CT)	SFA, VFA, SAT%	OS↑: SAT% (dichotomy: HR= 0.48; p< 0.01, continuous: HR =0.05; P< 0.01), SFA&VFA were negative, ; PFS↑: SAT% (dichotomy: HR = 0.32; p< 0.01, continuous: HR = 0.02; P< 0.01), SFA&VFA were negative,	Anti PD-1
Urothelial carcinoma	Martini (2021)	USA	70/49	69.5	20.1	L3 (CT)	SFI, VFI, IFI, body composition risk score*	OS: VFI was negative, SFI (low) (HR = 1.99; P = 0.043), IFI (low) (HR = 0.48; P = 0.018), body composition (High vs. Low HR = 6.72, P< 0.001/Intermediate vs. Low HR = 2.99, P = 0.029); PFS: SFI was negative, VFI (low) (HR = 1.76; P = 0.04), IFI (low) (HR = 0.48; P = 0.010), body composition risk score (High vs. Low HR = 5.82, P< 0.001/Intermediate vs. Low HR = 3.16, P = 0.005); CB: SFI and VFI were negative, IFI (low) (OR = 4.31; P = 0.036), body composition risk score (High vs. Low OR = 0.02, P = 0.003/Intermediate vs. Low OR = 0.11, P = 0.006);	ICI
	Yamamoto (2022)	Japan	31/22	74	5.7	L3 (CT)	FW/MFW/NW**	OS↓: MFW<FW<NW (p= 0.008); PFS↓: MFW<FW<NW (p<0.001)	Pembrolizumab (anti PD-1)
Melanoma	Sabel 2015	USA	48/32	56.7	NA	T12 to L4(CT)	Visceral fat distance	OS↓ (p = 0.022);	Ipilimumab (anti CTLA4)
	Hofmann 2019	German	147/	60	NA	L4 (CT)	TBF; SFA; VFA	PFS: all negative	Ipilimumab (anti CTLA4)
	Young 2020	USA	287/184	63	17.3	L3 (PET/CT)	TFI (tertile)	Response, OS and PFS: all negative (medium/high vs low)	anti-PD-1/PD-L1/anti CTLA4
	Faron(2021)	German	107/70	62	NA	L3/4 (CT)	VFI, SFI	OS: all negative	ICI
	Thaiss (2021)	German	18/10	61	NA	MRI	VATV, SATV	Responding patients showed limited variability of VATV and SATV	ICI
Head and neck squamous cell carcinoma	Takenaka (2022)	Japan	114/85	65	NA	L3 (CT)	SFI, VFI	RR↓: VFI (OR = 0.38,p< 0.05), SFI negative; OS↓: SFI (uni: HR = 1.82, p = 0.021/multi: HR = 1.14, p = 0.665), VFI (uni: HR = 2.51, p = 0.006/multi: HR = 1.93, p = 0.082); PFS: SFI negative and VFI (low) (uni: HR = 1.64, P = 0.044/Multi: HR = 2.07, P = 0.015); DCR: SFI negative	Nivolumaba (anti-PD-1)
Gastric cancer	Lin (2022)	China	101/79	62	23.1	L3 (CT)	VFI, ΔVFI, SFI, ΔSFI	Non-TR: all negative	Chemotherapy + ICI
Liver cancer	Xiao (2022)	China	172/149	51.4	9	L3 (CT)	VFI, SFI, VSR, TFI	OS↑: VFI (HR = 0.30, P = 0.001), SFI (HR = 0.31, P< 0.001), TFI (HR = 0.31, P< 0.001), VSR were negative; PFS: all negative	anti-PD-1/PD-L1
Metastatic solid tumor	Crombé 2020	France	117/62	63	NA	L3 (CT)	TFI, SFI, VFI, Δt-TFI, Δt-SFI, Δt-VFI	PFS↑: Δt-TFI (HR = 0.2, p< 0.0001), Δt-SFI (HR = 0.13, p = 0.001), Δt-VFI (HR = 0.09, P =0.0001), TFI, SFI, VFI were all negative	anti-PD-1, PD-L1, anti-PDL1 + anti-CTLA4
	Martini 2020	USA	90/53	NA	NA	L3 (CT)	SFI, IFI	OS↑: low risk (HR = 0.20, P< 0.001), intermediate risk was negative; PFS↑: low risk (HR = 0.38, P = 0.003), intermediate risk was negative; (low risk: SFI > =73 cm2/m2, Intermediate risk: SFI< 73 cm2/m2 and IFI< 3.4 cm2/m2 vs. high risk: SFI< 73 and IFI >= 3.4 cm2/m2)	ICI
	Esposito (2021)	Italy	153/62	58	27.9	L2/3 (CT)	TFA, VFA, SFA, VSR, Square root of VFA, Square root of SFA, Square root of VSR	OS↑: Square root of VSR (HR = 0.88, p = 0.047); others are negative	anti-PD-1/PD-L1

*Body composition risk score (in renal cell carcinoma) = IFI + 2*SM mean + SFI (favorable risk 0-1, intermediate risk 2, poor risk 3-4); Body composition risk score (in urothelial carcinoma) = SMI + 2 * attenuated SM mean + VFI (High risk 0-1, intermediate risk 2-3, Low risk 4).

**: NW (No Wasting; no post-therapeutic change in TAI and SMI); FW (Fat-Only Wasting; post-therapeutic decrease in TAI but no change in SMI); MFW (Muscle and Fat Wasting; post-therapeutic decrease in both TAI and SMI),

***: Response rate (RR) was defined as the percentage of patients who achieved complete response (CR) or partial response (PR); Disease control rate (DCR) was defined as the percentage of patients who achieved CR, PR, or stable disease.

CB, clinical benefit; CTLA-4, cytotoxic T lymphocyte associated protein 4; FBM, fat body mass; IFI, inter-muscular fat index; IMAC, intramuscular adipose content; irAEs, immune-related adverse events; ORR, objective response rate; OS, overall survival; PD-1, programmed cell death protein 1; PD-L1, programmed death ligand 1; PFS, progression-free survival; SCFM, subcutaneous fat mass; SFA, subcutaneous fat area; SFI, subcutaneous fat index; SMI, skeletal muscle index; TBF, total body fat; TFI, total fat index; TR, tumor remission; VATV, visceral adipose tissue volume; VFA, visceral fat area; VFM, visceral fat mass; VSR, visceral-to subcutaneous ratio; VFI, visceral fat index; WL, weight loss.

Adipose was measured by imaging-based biomarkers as follows. Total body fat (TBF) was defined as the sum of subcutaneous fat area (SCFA) and visceral fat area (VFA) according to the specific level of the body. Total fat index (TFI), visceral fat index (VFI), subcutaneous fat index (SFI) and inter-muscular fat index (IFI) were obtained by TBF, VFA, SCFA and inter-muscular fat area, respectively normalized for height squared. ΔVFI and ΔSFI represented the changes in VFI and SFI before and after neoadjuvant therapy, respectively. Δt-TFI, Δt-VFI and Δt-SFI were defined as the change of TFI, VFI and SFI from the first day of ICI initiation to early CT-scan evaluation (2 months later), divided by the time. Visceral fat volume (VATV) and subcutaneous fat volume (SATV) were measured by MRI for adipose tissue accumulation in each slice. Fat body mass (FBM), visceral fat mass (VFM) and subcutaneous fat mass (SCFM) was calculated as follows (22):


$$FBM\;=\;N_{fat}\;\times \;k_{fat}\;\times \;V_{voxel}\;\times \;\rho_{fat}.$$

$$VFM\;=\;N_{visceralfat}\;\times \;V_{voxel}\;\times \;\rho_{fat\;}\;.$$

SCFM = FBM – VFM

$$With\;k_{fat}=\frac{\bar{N_{fat\;}of\;the\;whole-body\;CT\;atlas}\;}{N_{fat}\;of\;truncated\;CT\;atlas}$$

**Figure 2:**
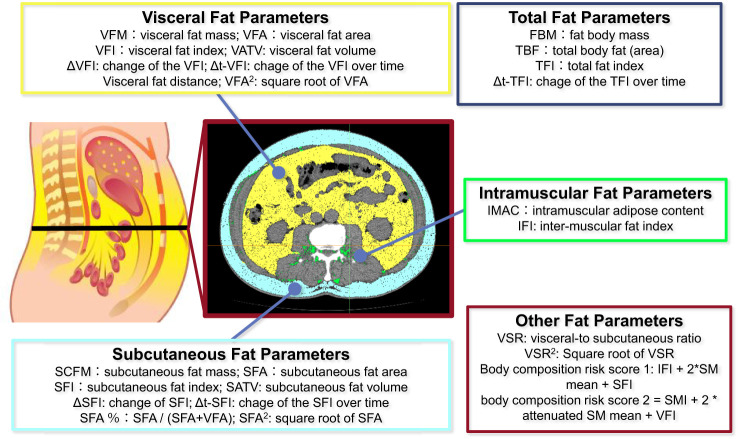
Summary of various fat parameters.

N_fat_ was defined as the number of voxels of fat, and V_voxel_ manifests the volume of one voxel (in ml). ρ_fat_ was equal to 0.923 g/ml. k_fat_ is calculated as the mean ratio of whole-body voxels of fat divided by numbers of voxels of fat between ischium and the eyes. Visceral-to subcutaneous ratio (VSR) was VFA/SFA ratio, while SAT% referred to SFA/(SFA + VFA) ratio. Body composition risk score used by DJ Martini et al., 2021 was calculated as s IFI + 2* skeletal muscle (SM) density mean + SFI (32), while body composition risk score adopted by another paper was calculated as skeletal muscle index (SMI) + 2 attenuated SM mean + VFI. Fat-only wasting represented a post-therapeutic decrease in TAI but no change in SMI, and muscle and fat wasting (MFW) represented a post-therapeutic decrease in TAI and SMI (29). Intramuscular adipose content (IMAC) was determined by the ratio of the attenuation in HU of the psoas muscle (21) and subcutaneous fat, or the ratio of the bilateral multifidus muscles density (21, 33) and subcutaneous fat density. Visceral fat distance was defined as the average distance between the anterior aspect of the vertebra and the linea alba along T12 to L4. These biomarkers could be summarized into five categories, including total fat, visceral fat, subcutaneous fat, inter-muscular fat and others, as is shown in **Figure 2**.

### 3.2 NSCLC

Overall, six papers reported on the correlation between body fat and ICI efficacy, in which three papers (22) demonstrated a positive correlation between more body fat and improved survival, while the other three papers failed to show such a relationship. Three studies reported on the metrics of visceral adipose tissue displayed controversial findings: one paper (22) illustrated the loss of visceral as a poor prognostic factor for patients’ OS, while the remaining two papers (21, 25) reported no significant association with survival. VFM was significantly demonstrated to be positively associated with patients’ 1-year OS (p=0.0075), while VFA and VFI failed to achieve a similar association. Two studies (22, 25) investigated the predictive value of subcutaneous adipose tissue but with opposite results: loss of SCFM in one study was regarded as a poor prognostic factor in one study, while failed to display correlation in the other study; SFI and SFA were also applied in one study but had no prognostic impact on OS. Intramuscular adipose tissue was reported by two papers (21, 34) both with no significant findings. VSR was employed in two studies (21, 25) that both demonstrated no predictive value of patients’ OS. Two papers (22, 25) investigated FBM and made opposite discoveries: one study reported it as a positive prognostic factor for OS in multivariate analysis, while the other paper found no significant association. TFI was reported in two papers (23, 25) with diverse findings: one study demonstrated loss of TFI as a positive prognostic factor for non-cachexia patients’ overall response rate (ORR) and PFS, whereas the other reported no significant association. One study (26) investigated weight loss of advanced NSCLC patients, which was significantly reflected by a loss of VAT and SAT, and showed a significant correlation with poorer OS (p<0.001).

### 3.3 Renal cell carcinoma (RCC)

Three studies in total reported the relationship between body fat and ICI efficacy with conflicting results: two papers with larger sample sizes (n=251 and n=79) demonstrated a positive correlation with survival, while one study reported no significant correlation. Subcutaneous adipose tissue was measured in two papers (27, 30) with diverse findings: SFI and SFA failed to show significant predictive value in these two studies, whereas SAT% was demonstrated as a positive prognostic factor for patients’ OS and PFS. One paper (27) investigated the predictive value of visceral adipose tissue using VFA but found no significant correlation with survival.TFI was showed in one study (32) to be significantly associated with OS and PFS, while the body composition risk score applied in the same study was a poor prognostic factor for OS, PFS and clinical benefit (CB).

### 3.4 Urothelial carcinoma (UC)

Two papers investigated the role of body fat in predicting survival outcomes after ICI treatment, both demonstrating it as a prognostic factor. One study (28) conducted on 70 patients with advanced urothelial carcinoma in the US reported a positive correlation of SFI and VFI with patients’ OS, PFS and CB, while IFI was associated with poorer survival outcomes. Body composition risk score was also demonstrated to be significantly associated with worse survival. In the other study (29), the best OS and PFS were observed in the NW group, followed by the FW and MFW groups, respectively.

### 3.5 Melanoma

Five studies investigated the relationship between body fat and ICI treatment outcomes, with conflicting results: two papers demonstrated a positive correlation, while three other papers failed to find a significant association with survival. Visceral fat was analyzed in four papers with inconsistent results: visceral fat distance was reported in one study (17) to significantly predict patients’ OS (p=0.022), while VFA displayed no predictive value for patients’ PFS in another study (35). VATV (36) and VAI (37) were also analyzed in separate studies, but no significant correlation with survival outcomes was found. The influence of subcutaneous adipose tissue was investigated in three papers (35–37) with similar results: all papers reported no association of subcutaneous fat with survival benefit measured by either SFA, SFI or SATV. Two studies (35, 38) investigated total adipose tissue, but neither TBF nor TFI displayed a significant association with PFS or OS.

### 3.6 HNSCC

Considering the therapeutic mode for HNSCC, only one paper (16) investigated the possible prognostic value of body fat on the survival benefit of recurrent or metastatic HNSCC patients. SFI was reported in this study to be positively associated with patients’ OS in univariate analysis, but no correlation was found with PFS or response rate (RR). Similarly, VFI was also demonstrated to have a significant association with better survival in terms of either OS, PFS or RR.

### 3.7 Gastric cancer

Only one paper (39) investigated the predictive value of body fat on ICI efficacy in patients with advanced gastric cancer. This retrospective study by Lin et al. involving 101 patients with locally advanced gastric cancer demonstrated no predictive value of VAI, SAI or variations of these two indexes on tumor remission.

### 3.8 Liver cancer

One paper (40) by L Xiao et al. reported on the correlation of body fat with the survival of 172 patients with primary liver cancer treated with anti-PD-1/PD-L1 therapy, and the results showed predictive effects of visceral fat, subcutaneous fat and total adipose tissue. VFI and SFI were both associated with better OS (p=0.001 and p<0.001) but not PFS. TFI also had a significant correlation with patients’ OS (p<0.001). Although VSR was applied, no significant findings were reported in terms of patients’ survival benefits.

### 3.9 Solid tumor

Three papers investigated the association of body fat and ICI therapy in various solid tumors altogether and found significant results in all papers. Visceral fat was reported in two of these studies (41, 42) with diverse findings: one paper applied TFI and its variation as the measurement for visceral fat and discovered a significant correlation between the variation of VFI and patients’ PFS (p=0.0001). VFA and its square root were employed in the other paper but reported no protective factor for patients’ OS in either of these indicators. All three papers measured subcutaneous adipose tissue as a possible prognostic factor but showed conflicting results: SFI was investigated in two papers (41, 43) with one regarding it as a protective factor for patients’ OS and OFS and the other reporting no significant correlation. The variation of SFI though displayed a positive correlation with patients’ PFS (p=0.001) in one study (41). SFA and its square root failed to show significant results in one paper (42). The influence of total body fat was investigated in two papers (41, 42) with conflicting results: only the variation of TFI demonstrated a positive association with patients’ PFS (p<0.0001) in one study, whereas no significant correlation was reported in TFI or total fat area (TFA). VSR and its square root were investigated in one paper (42), the latter of which was associated with better OS (p=0.047).

## 4 Discussion

Owing to the encouraging therapeutic effect on cancers, ICI has become an emerging treatment for cancers (13). Considering the variability of clinical outcomes of ICI therapy, it is urgent to identify patients that will get clinical benefits from it (44). Adipose tissue has been linked to prognosis in patients treated with immunotherapy, mainly including total body fat, visceral fat, subcutaneous fat and inter-muscular fat (6). However, the predictive value of these adipose biomarkers varies by cancer type. In this review, we summarize studies regarding the role of body fat composition in immunotherapy and its potential mechanisms.

Our systematic review identified seven cancers with clinical outcomes associated with imaging-based adipose biomarkers. A higher amount of total fat was correlated with favorable outcomes in RCC and liver cancer, while a higher amount of total fat was correlated with worse outcomes in NSCLC. More visceral adipose tissue predicted better clinical outcomes in NSCLC, UC, melanoma and liver cancer, whereas higher subcutaneous adipose accumulation was related to clinical benefits in NSCLC, RCC, UC and liver cancer. More inter-muscular adipose tissue was only associated with poorer survival benefits in UC. These varied results may be attributed to differences in structure, function and cytokine release of the different types of adipose tissue (45).

This systematic review provides a comprehensive overview of the imaging-based adipose biomarkers that can predict the clinical outcome of ICI therapy, offering insights into the use of radiometric adipose biomarkers. For lung cancer, FBM, TFI, VFM, SCFM and weight loss could be used as predictors of clinical outcome. For RCC, TFI, SAT% and body composition risk score integrating IFI, SM and SFI were the significant predictors of clinical outcome. For UC, the adoption of VFI, SFI and IFI to predict clinical outcomes may be effective. For melanoma, visceral fat distance manifested as a significant predictor. For HNSCC, VFI and SFI were selectable predictors. For gastric cancer, SFI was an optional predictor. For liver cancer, the significant predictors that could be adopted were VFI, SFI and VSR. When combining various cancer types, Δt-TFI, Δt-VFI, Δt-SFI, SFI and the square root of VSR could serve as potential predictors.

CT-based body composition is a promising predictive marker for cancer immunotherapy, superior to the traditional body indicator body mass index (BMI). BMI is a simple method of measuring body weight, typically calculated by dividing weight (in kilograms) by height (in meters) squared, and higher BMI was reported to be significantly associated with prognosis in various cancer studies (46). Nevertheless, BMI is limited by race specificity and individual heterogeneity (47),and cannot differentiate between different types of tissues like muscle and fat, or reflect body fat distribution (45). For example, individuals with a high BMI but low body fat may belong to the category of muscular obesity, while a person with a normal BMI but excessive visceral fat may have metabolic syndrome and related disease risks. Therefore, in clinical practice, BMI and body fat composition, among other indicators, should be considered in combination according to the specific circumstances to assess a patient’s health status and disease risks.

Body fat can impact immunotherapy through various mechanisms. As an endocrine organ, adipose tissue secretes adipokines such as leptin and adiponectin, which can contribute to tumor formation, invasion, angiogenesis, and immune evasion (48). Additionally, fat serves as a reservoir for immune cells, and mobilizing immune cells in fat can enhance anti-tumor activity (49). Higher fat levels may also provide patients with better nutrition and social status, potentially protecting them from cachexia (50). Moreover, tumor cells can also use fat to form abnormal tumor metabolic pathways to provide energy or evade immune surveillance (51). Tumor cells can also utilize fat to create abnormal metabolic pathways that supply energy and evade immune surveillance. We have reviewed relevant research on adipose tissue and identified several mechanisms by which it can influence the clinical outcome of ICI therapy.

Initially, adipose tissue was related to the pharmacokinetics, bioavailability and accumulation of the ICI. *In vivo* PET imaging found that the bio-distribution of ^9^Zr-nivolumab in adipose tissue was low, with uptake unaffected by the addition of 1 or 3 mg/kg. Patients with high FBM receive a relatively high dose in non-fat tissue, such as tumor targets. Bensch et al. also found that 1.56% to 18.95% of the injected dose accumulated in fat tissue (52). The “storage effect” of the adipose tissue can influence the overall distribution of therapeutic antibodies.

Additionally, obesity-related inflammation and pro-inflammatory cytokines can also have an impact on the immune response to ICI therapy. Chronic inflammation, characterized by elevated circulating levels of IL-1, IL-6 and TNF, is associated with both obesity and cancer (53). On the one hand, adipose tissue can have an adverse effect on immunotherapy, and the relationship between IL-1β and myeloid-derived suppressor cells (MDSCs) is of particular interest. MDSCs are a heterogeneous group of immature myeloid cells that promote immunosuppression and angiogenesis in tumors (54) and augment tumor growth through exacerbating inflammatory conditions. The recruitment of activated Treg lymphocytes and the function of CD4^+^ and CD8^+^ suppressed by interleukin-10, may contribute to the immunosuppressive effect of MDSCs (55). It has been reported that IL-1β, elevated by adipose tissue, induced the activation of MDSCs infiltrating tumor, and thus attenuate the therapeutic effect of ICI (23). Inhibition of IL-1β has been shown to improve tumor immunity (56). On the other hand, when cachexia is involved, characterized by adipose tissue loss, the effect of reduced adipose tissue loss may be obscured. It is reported that cancer cachexia is associated with reduced PD-1/PD-L1 inhibitor efficacy in NSCLC patients (57, 58). The failure to reduce IL-1β and cachexia-related mediators like TNF-α and IL-6 by reduced fat mass may contribute to this (59, 60).

Moreover, the close link between sone specific adipokines and cancer immunity could also serve as a potential explanation. Adipokines refer to the cytokines released from adipose tissue, including leptin, adiponectin and resistin. Leptin, the most well-studied adipokine in ICI therapy, increases the expression of PD-L1 on adipocytes during adipogenesis and promotes the immune escape of cancer (61). Nevertheless, high expression of PD-L1 in adipocytes, especially in visceral adipose tissue, also enhances the effectiveness of anti-PD-1/PD-L1 therapy. Tumor-infiltrating lymphocytes are also found to increase markers of exhaustion in diet-induced obese (DIO) mice, while progression rates in DIO mice are similar to control mice after anti-PD-L1 therapy (62). Adiponectin, another well-known adipocyte-secreted cytokine, is proven to negatively regulate the anti-tumor activity in CD8+ T cells through the activation of SATA3 (63). Resistin, a member of the resistin-like molecule family, was mostly found to influence the tumor itself. It is reported that resistin can elicit the expression of adhesion molecules including intercellular adhesion molecule-1 (ICAM-1) and vascular cell adhesion molecule (VCAM-1) to facilitate the cell invasion and metastasis of tumors (64). Besides, resistin also plays an important role in apoptosis-resistance, cancer stemness, angiogenesis and therapeutic resistance (65). Therefore, it is a double-edged sword that obesity promotes oncogenesis but with a robust host immune response to ICI.

There are also some limitations in this systematic review. Firstly, all studies included in this systematic review were retrospective. Although the studies reported have been fully included and discussed, the selection and heterogeneity of retrospective studies can’t be resolved. Hence, more prospective cohorts are needed to validate the association between fat and tumor immunotherapy. Secondly, there is a lack of universally agreed-upon tools to assess the risk of bias in included studies. Although the combination of NOS and RQS was used to assess the quality of the radiomics retrospective studies, it may not be sufficient for all types of studies. NOS was intended to detect the possible risk of bias in retrospective studies, while RQS mainly focuses on the guidance of good radiomics studies. Thirdly, the heterogeneity of adipose biomarkers used in the studies included in this review precluded the possibility of a quantitative meta-analysis, which limits the generalizability of the results. This limitation can commonly be seen in other reviews (66, 67). Meanwhile, the absence of confounding analysis, may potentially introduce a confounding effect that requires careful consideration. Finally, since the limited number of patients using ICI, a relatively emerging therapy of cancer, the population size in the studies included is relatively small. Besides, in some cancers like gastric cancer and head and neck squamous cell carcinoma, there are only a limited number of studies included, which means we should be more prudent when drawing conclusions regarding them. Therefore, more studies involving a large number of subjects are an unmet need in the future.

## 5 Conclusion

In summary, there is relatively well-supported evidence for imaging-based adipose biomarkers to predict the clinical outcome of ICI, despite the diversity of geographical populations and treatment protocols in each study. Generally, adipose tissue manifests a close correlation with clinical outcomes, with most of the studies included in our study showing positive outcome. Considering the diversity of imaging biomarkers and their corresponding outcomes, even in the same category, we summarize the significant predictors that have been proven by researches. Future studies should focus on the validation of these biomarkers in sufficiently large independent prospective cohorts.

## Data availability statement

The original contributions presented in the study are included in the article/**Supplementary Material**. Further inquiries can be directed to the corresponding authors.

## Author contributions

Conceptualization, XP, YX, YL, KN and HT. Data acquisition, XP and YX. Methodology, YL, LH and XY. Validation, LZ, MZ and XZ. Writing—original draft preparation, XP, YX. Writing—review and editing, XP, YL and KN. Visualization, KN and XP. Supervision, KN and HT. Project administration, XP, KN and HT. All authors contributed to the article and approved the submitted version.
