# Supplementary material for: Imaging-based adipose biomarkers for predicting clinical outcomes of cancer patients treated with immune checkpoint inhibitors: a systematic review
Source: Front Oncol. 2023 Oct 17;13:1198723. doi: 10.3389/fonc.2023.1198723 (PMC10616831; doi:10.3389/fonc.2023.1198723)
Supplement: Supplementary file 1 [file DataSheet_1.pdf]

frontiers

Supplementary Material

Imaging-Based Adipose Biomarkers for Predicting Clinical Outcomes of Cancer Patients Treated with Immune Checkpoint Inhibitors: A Systematic Review

Xinyu Pei†, Ye Xie†, Yixuan Liu†, Xinyang Cai, Lexuan Hong, Xiaofeng Yang, Luyao Zhang, Manhuai Zhang, Xinyi Zheng, Kang Ning*, Mengyuan Fang*, Huancheng Tang*

*** Correspondence: Huancheng Tang, 13412567043@163.com; Huancheng Tan, fangmengyuan186@163.com; Kang Ning, ningkang@sysucc.org.cn**

1 Supplementary Figures and Tables

1.1 Supplementary Tables

Supplementary Table 1. The search strategy of PubMed used in this systematic review (From inception to Feb 9, 2023)

PubMed		Number
#1	"Abdominal Obesity"[Title/Abstract] OR " Body Composition"[Title/Abstract] OR "Adipose Tissue"[Title/Abstract] OR "White Adipose Tissue"[Title/Abstract] OR "abdominal obesity"[Title/Abstract] OR "body composition"[Title/Abstract] OR "body fat"[Title/Abstract] OR "adipose tissue"[Title/Abstract] OR "abdominal fat"[Title/Abstract] OR "intraabdominal fat"[Title/Abstract] OR "subcutaneous fat"[Title/Abstract] OR "visceral adiposity"[Title/Abstract] OR "visceral obesity"[Title/Abstract] OR "visceral fat"[Title/Abstract] OR "perinephric fat"[Title/Abstract] OR "perirenal fat"[Title/Abstract] OR "lean body mass"[Title/Abstract] OR "lean soft tissue"[Title/Abstract] OR “fat” [Title/Abstract] OR “adiposity” [Title/Abstract]	443688
#2	"Immune checkpoint inhibition"[Title/Abstract] OR "immune checkpoint inhibitor"[Title/Abstract] OR "immune checkpoint inhibitors"[Title/Abstract] OR "immune checkpoint blocking"[Title/Abstract] OR "immune related"[Title/Abstract] OR immunotherapy[Title/Abstract] OR Atezolizumab[Title/Abstract] OR Tecentriq[Title/Abstract] OR Avelumab[Title/Abstract] OR Bavencio[Title/Abstract] OR Durvalumab[Title/Abstract] OR Imfinzi[Title/Abstract] OR Nivolumab[Title/Abstract] OR Opdivo[Title/Abstract] OR Ipilimumab[Title/Abstract] OR Yervoy[Title/Abstract] OR Pembrolizumab[Title/Abstract] OR Keytruda[Title/Abstract] OR Pidilizumab[Title/Abstract] OR Tremelimumab[Title/Abstract] OR Cemiplimab[Title/Abstract] OR "Anti PD L1"[Title/Abstract] OR "PD 1"[Title/Abstract] OR "programmed cell death protein 1"[Title/Abstract] OR "PD-L1"[Title/Abstract] OR "programmed death ligand 1"[Title/Abstract] OR "CTLA 4"[Title/Abstract] OR "cytotoxic T lymphocyte associated protein 4"[Title/Abstract] OR "Immunotherapy"[Mesh] OR "Antibodies, Monoclonal"[Mesh] OR "Antineoplastic Agents, Immunological"[Mesh] OR "avelumab" [Supplementary Concept] OR "atezolizumab" [Supplementary Concept] OR "Antineoplastic Agents, Immunological" [Pharmacological Action] OR "durvalumab" [Supplementary Concept] OR "pembrolizumab" [Supplementary Concept] OR "pidilizumab" [Supplementary Concept] OR "tremelimumab" [Supplementary Concept]	677852
#3	#1 AND #2	2044
#4	"predictor"[Title/Abstract] OR "predictors"[Title/Abstract] OR "predicting"[Title/Abstract] OR "responses"[Title/Abstract] OR "predictive"[Title/Abstract] OR "prognostic"[Title/Abstract]	4,166,876
#5	#3 AND #4	681

Supplementary Table 2. The search strategy of Embase used in this systematic review (From inception to Feb 9, 2023)

Embase		Number
#1	'white adipose tissue':ab,ti OR 'abdominal obesity':ab,ti OR 'body composition':ab,ti OR 'body fat':ab,ti OR 'adipose tissue':ab,ti OR 'abdominal fat':ab,ti OR 'intraabdominal fat':ab,ti OR 'subcutaneous fat':ab,ti OR 'visceral adiposity':ab,ti OR 'visceral obesity':ab,ti OR 'visceral fat':ab,ti OR 'perinephric fat':ab,ti OR 'perirenal fat':ab,ti OR 'lean body mass':ab,ti OR 'lean soft tissue':ab,ti OR 'fat':ab,ti OR 'adiposity':ab,ti	531661
#2	'immune checkpoint inhibition':ab,ti OR 'immune checkpoint inhibitor':ab,ti OR 'immune checkpoint inhibitors':ab,ti OR 'immune checkpoint blocking':ab,ti OR 'immune related':ab,ti OR 'immunotherapy':ab,ti OR 'atezolizumab':ab,ti OR 'tecentriq':ab,ti OR 'avelumab':ab,ti OR 'bavencio':ab,ti OR 'durvalumab':ab,ti OR 'imfinzi':ab,ti OR 'nivolumab':ab,ti OR 'opdivo':ab,ti OR 'ipilimumab':ab,ti OR 'yervoy':ab,ti OR 'pembrolizumab':ab,ti OR 'keytruda':ab,ti OR 'pidilizumab':ab,ti OR 'tremelimumab':ab,ti OR 'cemiplimab':ab,ti OR 'anti pd l1':ab,ti OR 'pd 1':ab,ti OR 'programmed cell death protein 1':ab,ti OR 'pd-l1':ab,ti OR 'programmed death ligand 1':ab,ti OR 'ctla 4':ab,ti OR 'cytotoxic t lymphocyte associated protein 4':ab,ti OR 'avelumab' OR 'atezolizumab' OR 'antineoplastic agents, immunological' OR 'durvalumab' OR 'pembrolizumab' OR 'pidilizumab' OR 'tremelimumab' OR 'immunological antineoplastic agent'/exp OR 'monoclonal antibody'/exp OR 'cancer immunotherapy'/exp	942312
#3	#1 AND #2	4054
#4	'predictor':ab,ti OR 'predictors':ab,ti OR 'predicting':ab,ti OR 'responses':ab,ti OR 'predictive':ab,ti OR 'prognostic':ab,ti	3092705
#5	#3 AND #4	729

Supplementary Table 3 . The quality of included cohort studies assessed by the Newcastle-Ottawa Quality Assessment Scale.

Category	Item	Option	G Popin et at. 2019	S Minami et al. 2020	C Baldessari et al. 2021	JH Degens et al. 2021	N Nishioka et al. 2022	FJ Bolte et al. 2022
Selection	Representativeness of the exposed cohort	truly representative of the citizen that have cancers and undergo ICI therapy in the community *	N	N	N	N	N	N
		somewhat representative of the citizen that have cancers and undergo ICI therapy in the community*	Y*	Y*	Y*	Y*	N	Y*
		selected group of users eg. nurses, volunteers	N	N	N	N	N	N
		no description of the derivation of the cohort	N	N	N	N	N	N
	Selection of the non exposed cohort	drawn from the same community as the exposed cohort*	Y*	Y*	Y*	Y*	Y*	Y*
		drawn from a different source	N	N	N	N	N	N
		no description of the derivation of the non exposed cohort	N	N	N	N	N	N
	Ascertainment of exposure	secure record (e.g., surgical records)*	Y*	Y*	Y*	Y*	Y*	Y*
		structured interview*	N	N	N	N	N	N
		written self report	N	N	N	N	N	N
		no description	N	N	N	N	N	N
	Demonstration that outcome of interest was not present at start of study	yes*	N	N	N	N	N	N
		no	Y	Y	Y	Y	Y	Y
Comparability	Comparability of cohorts on the basis of the design or analysis	study controls for the most important factor*	Y*	Y*	Y*	Y*	Y*	Y*
		study controls for any additional factor (This criteria could be modified to indicate specific control for a second important factor.) *	Y*	Y*	Y*	Y*	Y*	Y*
Outcome	Assessment of outcome	independent blind assessment *	N	N	N	N	N	Y*
		record linkage *	Y*	Y*	Y*	Y*	Y*	N
		self report	N	N	N	N	N	N
		no description	N	Y	N	N	N	N
	Was follow-up long enough for outcomes to occur (NOT IN CROSS SECTIONAL)	yes, it is an adequate follow up period for outcome of interest*	Y*	Y*	Y*	Y*	Y*	Y*
		no	N	N	N	N	N	N
	Adequacy of follow up of cohorts (NOT IN CROSS SECTIONAL)	complete follow up - all subjects accounted for *	N	N	N	N	N	N
		subjects lost to follow up unlikely to introduce bias - small number lost - >80 % (select an adequate %) follow up, or description provided of those lost *	N	Y*	N	N	N	Y*
		follow up rate < 80% (select an adequate %) and no description of those lost	N	N	N	N	N	N
		no statement	Y	N	Y	Y	Y	N
		Quality Score	7	8	7	7	6	7

Category	Item	Option	DJ Martini et al. 2021	Aslan et al. 2022	Wang et al. 2022	DJ Martini et al. 2021	S Yamamoto et al. 2022
Selection	Representativeness of the exposed cohort	truly representative of the citizen that have Mediterranean diet the community *	N	N	N	N	N
		somewhat representative of the citizen that have Mediterranean diet in the community*	Y*	N	Y*	Y*	Y*
		selected group of users eg nurses, volunteers	N	N	N	N	N
		no description of the derivation of the cohort	N	Y	N	N	N
	Selection of the non-exposed cohort	drawn from the same community as the exposed cohort*	Y*	Y*	Y*	Y*	Y*
		drawn from a different source	N	N	N	N	N
		no description of the derivation of the non-exposed cohort	N	N	N	N	N
	Ascertainment of exposure	secure record (e.g., surgical records)*	Y*	Y*	Y*	Y*	Y*
		structured interview*	N	N	N	N	N
		written self-report	N	N	N	N	N
		no description	N	N	N	N	N
	Demonstration that outcome of interest was not present at start of study	yes*	N	N	N	N	N
		no	Y	Y	Y	Y	Y
Comparability	Comparability of cohorts on the basis of the design or analysis	study controls for the most important factor*	Y*	Y*	Y*	Y*	Y*
		study controls for any additional factor (This criteria could be modified to indicate specific control for a second important factor.) *	Y*	Y*	Y*	Y*	Y*
Outcome	Assessment of outcome	independent blind assessment *	N	N	N	Y*	Y*
		record linkage *	Y*	Y*	Y*	Y*	Y*
		Self-report	N	N	N	N	N
		no description	N	N	N	N	N
	Was follow-up long enough for outcomes to occur (NOT IN CROSS SECTIONAL)	yes, it is an adequate follow up period for outcome of interest*	Y*	Y*	Y*	Y*	Y*
		no	N	N	N	N	N
	Adequacy of follow up of cohorts (NOT IN CROSS SECTIONAL)	complete follow up - all subjects accounted for *	N	N	N	N	N
		subjects lost to follow up unlikely to introduce bias - small number lost - >80 % (select an adequate %) follow up, or description provided of those lost) *	N	N	N	N	N
		follow up rate < 80% (select an adequate %) and no description of those lost	N	N	N	N	N
		no statement	Y	Y	Y	Y	Y
		Quality Score	7	6	7	8	8

Category	Item	Option	MS Sabel et al. 2015	KD Hofmann et al. 2019	AC Young et al. 2020	A Faron et al. 2021	WM. Thaiss et al. 2021
Selection	Representativeness of the exposed cohort	truly representative of the citizen that have Mediterranean diet the community *	N	N	N	N	N
		somewhat representative of the citizen that have Mediterranean diet in the community*	Y*	Y*	Y*	Y*	Y*
		selected group of users eg nurses, volunteers	N	N	N	N	N
		no description of the derivation of the cohort	N	N	N	N	N
	Selection of the non-exposed cohort	drawn from the same community as the exposed cohort*	Y*	Y*	Y*	Y*	Y*
		drawn from a different source	N	N	N	N	N
		no description of the derivation of the non-exposed cohort	N	N	N	N	N
	Ascertainment of exposure	secure record (e.g., surgical records)*	Y*	Y*	Y*	Y*	Y*
		structured interview*	N	N	N	N	N
		written self-report	N	N	N	N	N
		no description	N	N	N	N	N
	Demonstration that outcome of interest was not present at start of study	yes*	N	N	N	N	N
		no	Y	Y	Y	Y	Y
Comparability	Comparability of cohorts on the basis of the design or analysis	study controls for the most important factor*	Y*	Y*	Y*	Y*	N
		study controls for any additional factor (This criteria could be modified to indicate specific control for a second important factor.) *	Y*	Y*	Y*	Y*	N
Outcome	Assessment of outcome	independent blind assessment *	N	Y*	N	Y*	N
		record linkage *	Y*	Y*	Y*	Y*	Y*
		Self-report	N	N	N	N	N
		no description	N	N	N	N	N
	Was follow-up long enough for outcomes to occur (NOT IN CROSS SECTIONAL)	yes, it is an adequate follow up period for outcome of interest*	Y*	Y*	Y*	Y*	Y*
		no	N	N	N	N	N
	Adequacy of follow up of cohorts (NOT IN CROSS SECTIONAL)	complete follow up - all subjects accounted for *	N	N	N	N	N
		subjects lost to follow up unlikely to introduce bias - small number lost - >80 % (select an adequate %) follow up, or description provided of those lost) *	N	N	N	N	N
		follow up rate < 80% (select an adequate %) and no description of those lost	N	N	N	N	N
		no statement	Y	Y	Y	Y	Y
		Quality Score	7	8	7	8	5

Category	Item	Option	Y Takenaka et al. 2022	GT Lin et al. 2022	L Xiao et al. 2022	A Crombé et al. 2020	DJ Martini et al 2020	A Esposito et al. 2021
Selection	Representativeness of the exposed cohort	truly representative of the citizen that have cancers and undergo ICI therapy in the community *	N	N	N	N	N	N
		somewhat representative of the citizen that have cancers and undergo ICI therapy in the community*	Y*	Y*	Y*	Y*	Y*	Y*
		selected group of users eg. nurses, volunteers	N	N	N	N	N	N
		no description of the derivation of the cohort	N	N	N	N	N	N
	Selection of the non-exposed cohort	drawn from the same community as the exposed cohort*	Y*	Y*	Y*	Y*	Y*	Y*
		drawn from a different source	N	N	N	N	N	N
		no description of the derivation of the non-exposed cohort	N	N	N	N	N	N
	Ascertainment of exposure	secure record (e.g., surgical records)*	Y*	Y*	Y*	Y*	Y*	Y*
		structured interview*	N	N	N	N	N	N
		written self-report	N	N	N	N	N	N
		no description	N	N	N	N	N	N
	Demonstration that outcome of interest was not present at start of study	yes*	N	N	N	N	N	N
		no	Y	Y	Y	Y	Y	Y
Comparability	Comparability of cohorts on the basis of the design or analysis	study controls for the most important factor*	Y*	Y*	Y*	Y*	Y*	Y*
		study controls for any additional factor (This criteria could be modified to indicate specific control for a second important factor.) *	Y*	Y*	Y*	Y*	Y*	Y*
Outcome	Assessment of outcome	independent blind assessment *	N	N	N	Y*	N	Y*
		record linkage *	Y*	Y*	Y*	Y*	Y*	Y*
		Self-report	N	N	N	N	N	N
		no description	N	N	N	N	N	N
	Was follow-up long enough for outcomes to occur (NOT IN CROSS SECTIONAL)	yes, it is an adequate follow up period for outcome of interest*	Y*	Y*	Y*	Y*	Y*	Y*
		no	N	N	N	N	N	N
	Adequacy of follow up of cohorts (NOT IN CROSS SECTIONAL)	complete follow up - all subjects accounted for *	N	N	N	N	N	N
		subjects lost to follow up unlikely to introduce bias - small number lost - >80 % (select an adequate %) follow up, or description provided of those lost *	N	N	N	N	N	Y*
		follow up rate < 80% (select an adequate %) and no description of those lost	N	N	N	N	N	N
		no statement	Y	Y	Y	Y	Y	N
		Quality Score	7	7	7	8	7	8

Supplementary table 4. Radiomics Quality Score

Reference	G Popin et at. 2019	S Minami et al. 2020	C Baldessari et al. 2021	JH Degens et al. 2021	N Nishioka et al. 2022	FJ Bolte et al. 2022	DJ Martini et al. 2021	Aslan et al. 2022	Wang et al. 2022	DJ Martini et al. 2021	S Yamamoto et al. 2022
Total	13	3	5	12	5	5	6	4	5	6	6
Image protocol quality	2	2	2	2	2	2	2	2	2	2	2
Multiple segmentations	0	0	0	0	0	1	0	0	0	1	1
Phantom study	1	0	0	0	0	0	1	0	1	0	0
Multiple time points	0	0	0	1	1	0	0	0	0	1	1
Feature reduction	3	3	3	3	3	3	3	3	3	3	3
Non-radiomics features	1	1	1	1	1	1	1	1	1	1	1
Relation with phenotype	0	0	1	1	1	0	0	0	1	0	0
Cut-off analysis	1	1	1	1	1	1	1	1	1	1	1
Discrimination	1	0	1	0	0	0	1	0	0	1	0
Calibration statistics	1	1	1	1	1	1	1	1	1	1	1
Prospective study	0	0	0	0	0	0	0	0	0	0	0
Validation	3	-5	-5	3	-5	-5	-5	-5	-5	-5	-5
Gold standard	0	0	0	0	0	0	0	0	0	0	0
Clinical utility	0	0	0	0	0	0	0	0	0	0	0
Cost-effectiveness	0	0	0	0	0	0	0	0	0	0	0
Open data	0	0	0	0	0	1	1	1	0	0	1

Reference	MS Sabel et al. 2015	KD Hofmann et al. 2019	AC Young et al. 2020	A Faron et al. 2021	WM. Thaiss et al. 2021	Y Takenaka et al. 2022	GT Lin et al. 2022	L Xiao et al. 2022	A Crombé et al. 2020	DJ Martini et al 2020	A Esposito et al. 2021
Total	3	5	4	7	5	4	5	3	5	4	5
Image protocol quality	2	2	2	2	2	2	2	2	2	2	2
Multiple segmentations	0	1	0	1	0	0	0	0	1	0	1
Phantom study	0	0	1	0	0	0	1	0	0	0	0
Multiple time points	0	0	0	1	1	0	1	0	1	0	0
Feature reduction	3	3	3	3	3	3	3	3	3	3	3
Non-radiomics features	1	1	1	1	1	1	1	1	1	1	1
Relation with phenotype	0	0	0	1	0	0	0	0	0	0	0
Cut-off analysis	1	1	1	1	1	1	1	1	1	1	1
Discrimination	0	0	0	0	0	1	0	0	0	1	0
Calibration statistics	1	1	1	1	1	1	1	1	1	1	1
Prospective study	0	0	0	0	0	0	0	0	0	0	0
Validation	-5	-5	-5	-5	-5	-5	-5	-5	-5	-5	-5
Gold standard	0	0	0	0	0	0	0	0	0	0	0
Clinical utility	0	0	0	0	0	0	0	0	0	0	0
Cost-effectiveness	0	0	0	0	0	0	0	0	0	0	0
Open data	0	1	0	1	1	0	0	0	0	0	1

Supplementary Table 5. The details of ICI treatment in each study.

Cancer(s)	Author (year)	Administration	Number of times /Frequency	Study Duration	Dose
Lung cancer	Popin (2019)	-	every 14 days	-	3 mg/kg
	Minami (2020)	-	-	-	-
	Baldessari (2021)	-	More than one infusion of pembrolizumab	between July 2017 and December 2018	-
	Degens (2021)	intravenous infusion	every 2 weeks	between June 2015 and December 2018	-
	Nishioka (2022)	-	-	between May 2016 and December 2018	-
	Bolte (2022)	-	-	between 2015 and 2021	-
Renal cell carcinoma	Martini (2021)	-	at least 1 dose of ICI	2015-2020	-
	Aslan (2022)	-	-	between October 2010 and October 2021	-
	Wang (2022)	-	-	from October 2015 through April 2021	-
Urothelial carcinoma	Martini (2021)	-	-	from 2015 to 2020	-
	Yamamoto (2022)	-	-	-	-

Melanoma	Sabel 2015	-	-	-	-
	Hofmann 2019	-	-	between August 2010 and April 2017	-
	Young 2020	-	-	-	-
	Faron(2021)	-	-	between January 2013 and August 2019	-
	Thaiss (2021)	-	-	Between 09/2014 and 10/2016	-
Head and neck squamous cell carcinoma	Takenaka (2022)	-	-	between 2017 and 2020	-
Gastric cancer	Lin (2022)	intravenously	on the first day of chemotherapy, along with the circle	from January 2019 to April 2021	according to the patient's body surface area
Liver cancer	Xiao (2022)	-	-	between August 2018 and October 2020	-
Metastatic solid tumor	Crombé 2020	-	-	-	-
	Martini 2020	-	-	from 2009 through 2017	-
	Esposito (2021)	-	-	between August 2014 and May 2019	-

Supplementary Table 6. Number of studies regarding each biomarker in each cancer type.

Biomarkers	Lung Cancer	Renal cell carcinoma	Urothelial carcinoma	Melanoma	Head and neck squamous cell carcinoma	Gastric cancer	Liver cancer	Metastatic solid tumor
VFM	1							
VFA	2	1		1				1
VFI	2		1	1	1	1	1	1
VSR	2						2	1
Visceral fat distance				1				
VATV				1				
ΔVFI						1		
Δt-VFI								1
Square root of VFA								1
Square root of VSR								1
SFI	2	1	1	1	1	1	1	2
SCFM	2							
SFA	1	1		1				1
SATV				1				
SAT%		1						
ΔSFI						1		

Δt-SFI								1
Square root of SFA								1
TFI	2	1		1			1	1
Δt-TFI								1
TBF				1				
TFA								1
IMAC	2							
IFI			1					1
FBM	2							
FW/MFW/NW			1					
Body composition risk score		1	1					
